# Type 2 diabetes exaggerates exercise effort and impairs exercise performance in older women

**DOI:** 10.1136/bmjdrc-2015-000124

**Published:** 2015-09-30

**Authors:** A G Huebschmann, W M Kohrt, L Herlache, P Wolfe, S Daugherty, J EB Reusch, T A Bauer, J G Regensteiner

**Affiliations:** 1 Department of Medicine; Division of General Internal Medicine, University of Colorado (CU) School of Medicine (SOM), Aurora, Colorado, USA; 2 Center for Women's Health Research, Aurora, Colorado, USA; 3 Division of Geriatrics, Aurora, Colorado, USA; 4 CU-SOM Department of Biostatistics, Aurora, Colorado, USA; 5 Division of Cardiology, Aurora, Colorado, USA; 6 Division of Endocrinology, Aurora, Colorado, USA

**Keywords:** Type 2 Diabetes, Exercise Habits, Physical Activity Epidemiology, Behavior Research

## Abstract

**Objective:**

Type 2 diabetes mellitus (T2DM) is associated with high levels of disability and mortality. Regular exercise prevents premature disability and mortality, but people with T2DM are generally sedentary for reasons that are not fully established. We previously observed that premenopausal women with T2DM report greater effort during exercise than their counterparts without diabetes, as measured by the Rating of Perceived Exertion (RPE) scale. We hypothesized that RPE is greater in older women with T2DM versus no T2DM.

**Research design and methods:**

We enrolled overweight, sedentary women aged 50–75 years with (n=26) or without T2DM (n=28). Participants performed submaximal cycle ergometer exercise at 30 W and 35% of individually-measured peak oxygen consumption (35% VO_2_peak). We assessed exercise effort by RPE (self-report) and plasma lactate concentration.

**Results:**

VO_2_peak was lower in T2DM versus controls (p=0.003). RPE was not significantly greater in T2DM versus controls (30 W: Control, 10.4±3.2, T2DM, 11.7±2.3, p=0.08; 35% VO_2_peak: Control, 11.1±0.5, T2DM, 12.1±0.5, p=0.21). However, lactate was greater in T2DM versus controls (p=0.004 at 30 W; p<0.05 at 35% VO_2_peak). Greater RPE was associated with higher lactate, higher heart rate, and a hypertension diagnosis (p<0.05 at 30 W and 35% VO_2_peak).

**Conclusions:**

Taken together, physiological measures of exercise effort were greater in older women with T2DM than controls. Exercise effort is a modifiable and thereby targetable end point. In order to facilitate regular exercise, methods to reduce exercise effort in T2DM should be sought.

**Trial number:**

NCT00785005.

**Key messages**

- A novel barrier to physical activity that we have identified previously is that exercise feels more difficult to sedentary premenopausal women with type 2 diabetes mellitus (T2DM) than their similarly obese and sedentary counterparts without diabetes.
- We found statistically significant differences in plasma lactate during low- to moderate-intensity exercise in postmenopausal women with T2DM, as compared to their counterparts without diabetes; we also found clinically meaningful differences in exercise effort as measured subjectively by the Borg Rating of Perceived Exertion (RPE).
- An important take-home point for clinicians is to encourage patients to be physically active at a pace that is personally comfortable, as this tends to be associated with both good adherence and a good physiological fitness response.

The prevalence of type 2 diabetes mellitus (T2DM) continues to rise worldwide and the highest prevalence rates are found among older adults.^1^ Exercise is considered a critical cornerstone of treatment for people with T2DM due to its beneficial effects on glycemic control, physical fitness, cardiovascular health and prevention of disability as well as premature mortality.^2–4^ However, people with T2DM are consistently more sedentary than similarly obese people who do not have diabetes, for reasons that are not clear.^5^ ^6^ Since lower fitness levels are linked to cardiovascular morbidity and mortality in populations with and without T2DM,^7^ ^8^ understanding and overcoming barriers to physical activity for people with T2DM is critically important.

A novel barrier to physical activity that we have identified previously is that exercise feels more difficult to sedentary people with T2DM than their similarly obese and sedentary counterparts without diabetes.^9^ ^10^ Specifically, we have shown that effort during low intensity exercise is greater in premenopausal women with T2DM versus similarly obese controls, as measured by both the Borg Rating of Perceived Exertion (RPE) and plasma lactate concentrations during exercise. Since RPE is modifiable^11–13^ and plays a significant role in adherence to prescribed physical activity,^14–16^ it has great potential relevance as a modifiable barrier to physical activity. In addition, these findings may partly explain some barriers to physical activity identified in prior questionnaires and focus groups of people with T2DM, such as ‘difficulty keeping up with others who don't have T2DM.’^17^ Finally, RPE is related to the affective response to exercise,^18^ ^19^ and the 2010 American Diabetes Association physical activity guidelines suggest that “affective responses to exercise may be important predictors of physical activity adoption and maintenance.”^3^

Important possible mediators of increased exercise effort include impaired cardiorespiratory fitness levels and submaximal exercise responses. Submaximal exercise impairments include a slowed VO_2_ uptake kinetics response that represents a delay in achieving steady-state oxygen utilization during constant work rate exercise. Among adolescents and middle-aged adults, participants with T2DM have significantly worse peak cardiorespiratory fitness levels and worse submaximal exercise performance than their counterparts without diabetes.^10^ ^20–22^ Less is known about these measures of exercise performance in older adults with T2DM^23^ even though older adults have the highest prevalence of diabetes.^1^ Therefore, we sought to compare measures of exercise effort and exercise performance during exercise in older women with T2DM versus their counterparts without diabetes. We hypothesized that there would be greater perceived effort during low to moderate intensity exercise in participants with T2DM compared to controls without diabetes. We also hypothesized that both fitness levels and submaximal exercise responses would be more impaired in participants with T2DM than controls, despite recruiting participants of similar weight and similar levels of habitual physical activity. We studied women with T2DM because T2DM confers greater exercise impairment in women than men.^24^

## Research design and methods

In this cross-sectional study, we enrolled overweight, sedentary women with or without T2DM from the metropolitan Denver, Colorado, USA community between 2007 and 2011. Participants were 50–75 years old with either T2DM (n=26) or controls without diabetes (n=28). We defined people without diabetes by the prevailing American Diabetes Association guidelines at the time of the research (ie, normal fasting glucose <100 mg/dL and glycated hemoglibin, HbA1c <6%).^25^ In addition, to minimize finding insulin resistance in control participants, we excluded controls with >1 first-degree relative with T2DM.^25^ Additional inclusion criteria ensured similar overweight and sedentary status in study groups: overweight/obese body mass index (BMI) (25–39.9 kg/m^2^) and self-report of leisure physical activity behavior of <60 min/week. Participants were postmenopausal as documented by no menses in >12 months and by measured follicle-stimulating hormone levels.^10^ Our exclusion criteria included conditions that imposed safety concerns or could impair exercise performance, such as uncontrolled hypertension, a history of atherosclerosis, congestive heart failure, autonomic or peripheral neuropathy, chronic disease of the lung, liver or kidneys, microalbuminuria, or tobacco use within 1 year.^26^ In addition, we excluded participants with prolonged duration of T2DM or suboptimal disease control: HbA1c >8.5%, duration of diabetes >20 years, microalbuminuria (urine microalbumin/creatinine >30).^27^ Use of insulin, thiazolidinediones, GLP-1 agonists and DPP-4 inhibitors was excluded since these drugs might either affect exercise capacity or suggest more advanced disease.^22^ ^28^

### Exercise effort

Measures of exercise effort included RPE (primary outcome) and plasma lactate concentration (secondary outcome) measured during eight bouts of constant work rate submaximal exercise. The Borg RPE scale is the gold standard measure of RPE during exercise in healthy^29^ ^30^ and diabetic populations.^31^ The range of scores on this ordinal scale is from 6 to 20 with verbal anchors every two points (eg, RPE=11, ‘light’, RPE=13, ‘somewhat hard’). To minimize bias, participants and the research staff recording RPE were blinded to the work rate. Plasma lactate concentration was measured using the lactate dehydrogenase method on blood drawn in perchloric acid tubes.^10^ RPE and lactate concentration were measured on two separate study dates at both an absolute work rate (30 W, 4 bouts) and a relative work rate (4 bouts). We designed the relative work rate to be 35% of the VO_2_peak from each participant's peak exercise test, in order to account for the influence of cardiorespiratory fitness on exercise effort. We chose 35% VO_2_peak as the relative work rate to ensure participants achieved a steady-state VO_2_ level that was reliably below yet close to the lactate threshold. In our prior published work in premenopausal women, 35% VO_2_peak was ∼30 W for participants with T2DM.^9^

### Nutritional and body composition assessments prior to exercise testing

To control for effects of diet on exercise performance, participants consumed a eucaloric study diet with standardized macronutrient distributions for 3 days prior to exercise testing and fasted for at least 4 h prior to exercise testing.^10^ Eucaloric diets were developed by registered dieticians based on body composition assessed by Dual-energy X-ray Absorptiometry (DXA scan, Hologic/Discovery W, Hologic Inc, Bedford, Massachusetts, USA). DXA was also used to assess the exercise effort-related covariate of total fat-free mass.

### Exercise testing

The exercise testing procedures have been described in detail previously.^10^ ^32^ In brief, each exercise test began with the participant seated upright at rest for 3 min on the cycle ergometer (Lode ergometer, MedGraphics, Minneapolis, Minnesota, USA) breathing into a mouthpiece connected to a metabolic cart (Ultima CPX, MedGraphics). Participants first performed a familiarization graded cycle ergometer exercise test (GXT). On a subsequent day, we used a ramping protocol GXT to measure VO_2_peak. During the ramping protocol GXT, the rate of increase of the work rate to peak exercise capacity was individualized to ensure an optimal test duration of 10–14 min (eg, if the familiarization GXT peak work rate was 55 W, the wattage increased continuously by 0.0833 W/s to reach a work rate of 55 W at 11 min). During exercise, VO_2_ was measured breath-by-breath and time-averaged over 30 s intervals. We defined VO_2_peak by standard convention as the peak VO_2_ associated with an RER ≥1.1 or as a VO_2_ plateau despite an increase in workload.^33^

#### Exercise performance measures and other predictors of exercise effort

Our secondary outcomes of exercise performance included cardiorespiratory fitness (VO_2_peak) and a measure of submaximal exercise response (time constant (τ_2_) from VO_2_ uptake kinetics) at both 30 W and 35% VO_2_peak.

### Kinetics measurements during constant-load exercise

On subsequent study dates, participants performed eight bouts of submaximal constant-load exercise with work rates alternating between 30 W and 35% VO_2_peak. Each exercise bout was 8 min in duration. The research assistants recording the data and participants were blinded to the work rate. During constant-load exercise, we assessed submaximal exercise response using VO_2_ kinetics, where a longer time constant (τ_2_) represents a longer time to achieve steady-state VO_2_.^10^ As previously described, for each work rate, gas-exchange data for kinetic analyses were processed using a software program developed in our laboratory.^10^ ^32^ The pulmonary VO_2_ kinetic responses data for each of the bouts of oxygen consumption were evaluated using a two-component exponential model.^26^

### Physiological predictors of exercise effort

**Figure 1:**
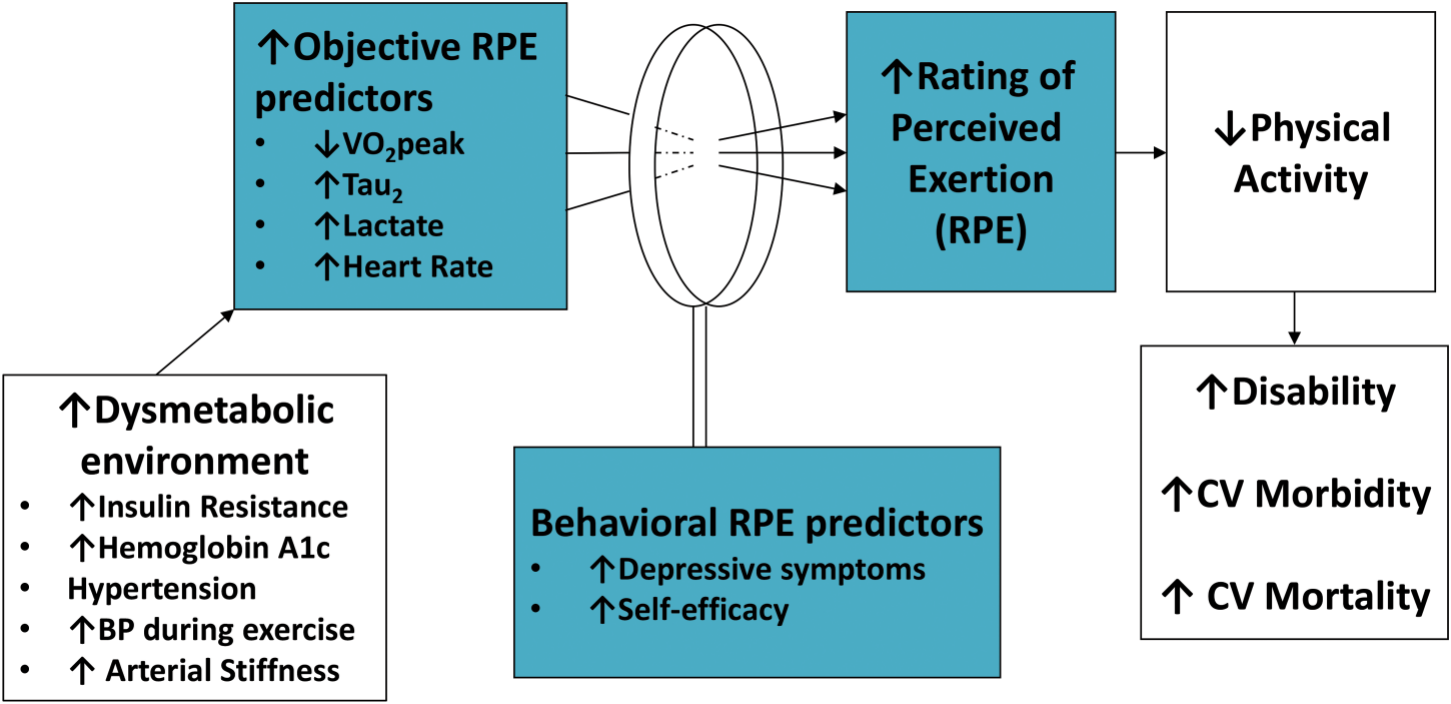
Conceptual model of perceived effort during exercise.

According to our conceptual model (figure 1), we considered the following physiological variables as potential predictors of exercise effort: heart rate during exercise, VO_2_peak and τ_2_. In addition, we assessed metabolic and vascular factors that we hypothesized were likely to impair the physiological response to exercise in people with T2DM: Homeostasis Model Assessment of Insulin Resistance (HOMA-IR),^34^ markers of endothelial dysfunction such as a diagnosis of hypertension and arterial stiffness expressed as pulse-wave velocity^35^ (SphygmaCor CP system, AtCor Medical). HOMA-IR was calculated per standard convention from blood glucose and insulin measurements collected during a 12 h fast.^34^ We also measured glucose levels during the 8 min of exercise as an additional potential predictor of effort. A diagnosis of hypertension was obtained by self-report and validated during the medical history and physical examination by the study physician/PI (AGH). Pulse-wave velocity was assessed after a 4 h fast and prior to any exercise testing performed in the same study visit.

### Behavioral predictors of exercise effort

According to our conceptual model where behavioral factors may influence the interpretation of physiological cues (figure 1), we also considered the following behavioral variables as potential predictors of RPE: self-efficacy to perform physical activity^36–38^ and depressive symptoms.^39^ ^40^ We assessed these behavioral variables by paper surveys conducted prior to any exercise testing performed in the same study visit.

### Power calculation

Based on our preliminary data where we observed an RPE difference of 1.3±1.4 (mean±SD) between the T2DM and overweight control groups,^9^ we estimated that 54 participants would provide 82% power to detect a between-group difference in RPE of 0.8 in the 8 min of exercise at the 0.05 level. We considered a between-group RPE difference of one point to be clinically meaningful.^41^

### Statistical analysis

All outcome and predictor variables were examined with descriptive statistics and graphic summaries, overall and by T2DM status. To determine group differences in RPE and lactate, 30 W data collected during four separate bouts of exercise and 35%VO_2_peak data collected during four separate bouts of exercise were evaluated using separate maximum likelihood repeated measures models (eg, 30 W model assessed 4 repeated measures).^42^ The primary outcomes were RPE and lactate at minute 8. We also assessed changes in RPE within and between-groups across minutes 2, 4, 6 and 8 at 30 W and 35% VO_2_peak to determine if RPE levels were at steady-state, defined as equivalent levels at minutes 6 and 8, and to compare the RPE change from minutes 2 to 8 by study group. We also assessed lactate concentrations at rest prior to exercise and the change in lactate concentrations from the resting baseline to the 8 min of exercise (Δlactate), by using separate maximum likelihood repeated measures models. Finally, we compared group differences in heart rate, also using a maximum likelihood repeated measures model at each of 2, 4, 6 and 8 min into the exercise bouts. To ensure that our data at the relative work rate of 35% VO_2_peak was accounting for fitness levels appropriately, we also conducted a sensitivity analysis to estimate group differences in effort variables (ie, RPE, lactate during minute 8, heart rate during minute 8 of exercise) with adjustment for fitness at the 30 W absolute work rate for comparison with the group differences from the relative work rate of 35% VO_2_peak that accounted for fitness. We also conducted a sensitivity analysis to estimate group differences in resting lactate concentration with adjustment for fitness.

Continuous variables that were only measured once were compared with a two-sided two-sample t test, and dichotomous variables were compared with either a χ^2^ test for equal proportions or Fisher's exact test.

Secondary analyses were conducted to evaluate potential covariates according to our conceptual model (figure 1). We first estimated Pearson's product-moment correlation coefficients between candidate predictor variables to rule out excessive collinearity (r>0.8). We then estimated Pearson's correlations between the effort variables, RPE and lactate and the potential covariates. Predictor variables with a correlation coefficient ≥0.2 were added to the maximum likelihood models described above; we retained predictor variables in the final model when p<0.05. For groupings of predictor variables with relatively high collinearity (eg, metabolic predictors of a diagnosis of T2DM (dichotomous variable), HOMA and HbA1c), we selected the metabolic predictor that created the best fitting model based on the BIC statistic.

All analyses were conducted in SAS V.9.2.^43^

## Results

### Study population

We consented 97 women for participation, 35 women with T2DM and 62 control women without diabetes. After screening laboratory testing and procedures, we excluded 43 women who met exclusion criteria, including 18 women with prediabetes. Women in the T2DM (n=26) and control groups without diabetes (n=28) were similar in age, BMI and baseline physical activity levels, as per study design (table 1). Hypertension prevalence was ∼50% in each study group. As compared with the control group, the T2DM group included more non-white women (p=0.01) and had higher mean HbA1c levels (p<0.001).

**Table 1:** Participant characteristics

Variable	Overweight control (n=28)	Type 2 diabetes (T2DM) (n=26)	p Value
Duration of T2DM, years (SD)	NA	5.1 (5.0)	NA
Age, years (SD)	59.8 (5.8)	59.3 (5.7)	0.39
Ethnicity, n (% Hispanic)	1 (4)	3 (12)	0.34
Race, n (% Caucasian)	27 (96)	18 (67)	0.01
Hypothesized physiological RPE predictors			
BMI, kg/m^2^ (SD)	30.1 (2.8)	31.3 (3.9)	0.25
HbA1c, %	5.7 (0.3)	6.8 (0.6)	<0.001
Homeostasis Model Assessment of Insulin Resistance (HOMA-IR)	4.0 (1.9)	6.7 (4.6)	0.01
HTN prevalence (%)	46	50	0.99
Pulse wave velocity (m/s)	9.2 (0.7)	9.9 (0.8)	0.06
Reported usual physical activity, MET h/week (SD)	222 (35)	231 (25)	0.32
VO_2_peak (mL/min)	1470 (286)	1313 (244)	0.02
VO_2_peak (mL/kg/min)	17.8 (3.0)	15.4 (2.5)	0.003
Work rate during 35% VO_2_peak (SD)	43.5 (7.7)	35.9 (7.1)	<0.0001
Heart rate during 8 min of exercise at 30 W (bpm)	91 (2)	102 (2)	0.002
Heart rate during 8 min of exercise at 35% VO_2_peak	98 (3)	105 (3)	0.04
Resting lactate concentrations (mmol/L)	0.39 (0.03)	0.65 (0.03)	<0.0001
Lactate during 8 min of exercise at 30 W (mmol/L)	0.76 (0.10)	1.18 (0.10)	0.004
Lactate at 8 min at 35% VO_2_peak (mmol/L)	0.99 (0.10)	1.28 (0.10)	0.047
τ_2_ at 30 W	39.2 (13)	36.2 (9.1)	0.34
τ_2_ at 35% VO_2_peak	38.6 (12.2)	38.5 (10.2)	0.98
Hypothesized behavioral RPE predictors			
Depressive symptoms			
CES-D	11.4 (3.7)	11.7 (5.6)	0.77
PHQ-9	3.3 (3.3)	3.2 (3.2)	0.96
Self-efficacy scores			
Endurance self-efficacy	746 (359)	739 (264)	0.94
Lorig self-efficacy	481 (95)	479 (94)	0.92
Sallis self-efficacy	45 (8)	47 (8)	0.23

Data expressed as mean (SE) for heart rate, lactate; data expressed as mean (SD) for all other continuous variables.

Missing data for <10% of participants with the exception of pulse wave velocity where we are missing 40% of data for each study group.

BMI, body mass index; CES-D, Center for Epidemiological Studies Depression; HbA1c, glycated hemoglobin; HTN, hypertension; PHQ-9, Patient Health Questionnaire-9; RER, respiratory exchange ratio; T2DM, type 2 diabetes mellitus.

### Markers of effort—RPE and lactate

**Figure 2:**
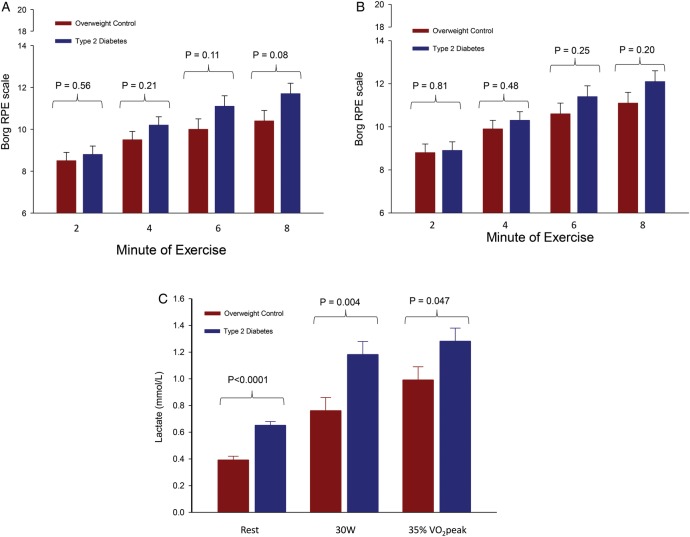
(A) Group differences in rating of perceived exertion over time at 30 W. (B) Group differences in rating of perceived exertion over time at 35% VO_2_peak. (C) Higher lactate concentrations in T2DM during rest and exercise.

At both absolute work rates and relative work rates that accounted for fitness differences among participants, RPE was greater, but not significantly so, in the T2DM versus control group at minutes 2, 4, 6 and 8 of exercise (figure 2A, B). Of note, despite all participants reaching steady-state oxygen uptake levels within the first 2 min of exercise, the RPE levels continued to rise during 8 min of exercise and had not yet reached steady-state at 8 min of exercise (the RPE slope was different from zero (p<0.001) from minutes 6 to 8 in each study group). The change in RPE from minute 2 to 8 was greater, but not significantly so, in the T2DM group compared to the control group (p=0.07).

Lactate concentrations are shown in figure 2C. To summarize, mean lactate levels during minute 8 of exercise were significantly greater in the T2DM group as compared with control women (p=0.004 at 30 W and p=0.046 at 35% VO_2_peak, figure 2C). Mean lactate levels were also significantly greater at rest in the T2DM group compared with control women (p<0.0001). The difference between resting and exercise lactate concentrations (Δlactate) was not significantly greater in the T2DM group compared with control women (30 W Δlactate: 0.53±0.08 vs 0.37±0.08 (p=0.17); 35% VO_2_peak Δlactate: 0.62±0.08 vs 0.60±0.08, p=0.86). Our sensitivity analysis showed that group differences in resting lactate remained significantly different after adjustment for VO_2_peak (p<0.001), and we observed no statistical association between VO_2_peak and resting lactate concentration (p=0.98).

### Physiological effort-related variables

VO_2_peak was significantly lower in the T2DM group versus study group without diabetes by absolute and weight-adjusted measures (table 1). However, τ_2_ was not different between study groups at 30 W or 35% VO_2_peak (table 1). Of the physiological variables that were prespecified as candidate predictors of RPE (figure 1), those that were significantly different between study groups included heart rate during exercise, lactate levels during exercise, VO_2_peak, HbA1c and HOMA-IR (table 1). In a sensitivity analysis adjusting for fitness levels as VO_2_peak (mL/min) rather than accounting for fitness differences at the relative work rate of 35% VO_2_peak, mean lactate levels and heart rate during exercise at 30 W remained significantly greater in the T2DM group as compared with control women (p=0.02 for lactate; p=0.01 for heart rate).

### Association of physiological variables with effort

In the regression model assessing predictors of RPE in the 8th min of exercise with adjustment for insulin resistance by HOMA-IR, the physiological variables significantly associated with RPE were heart rate during exercise, diagnosis of hypertension and plasma lactate during exercise (table 2). Although HOMA-IR was not a statistically significant predictor of RPE, it was included in the model as a precision efficiency variable (ie, the model fit was enhanced by including HOMA-IR, and HOMA-IR outperformed the other metabolic candidate variables of T2DM diagnosis, glucose level during exercise and HbA1c). In the regression model assessing predictors of lactate level at minute 8, the only significant predictor at both 30 W and 35% VO2peak was heart rate during exercise. At the 30 W work rate only, τ_2_ was also significantly associated with lactate.

**Table 2:** Regression models for predictors of RPE and lactate at 30 W and at 35% VO_2_peak

Effort predictor variables	30 W F statistic (p value)	35% VO_2_peak F statistic (p value)
Physiological RPE predictor model		
Heart rate during 8 min exercise	9.8 (p=0.002)	4.1 (p=0.045)
Hypertension diagnosis (yes/no)	6.4 (p=0.02)	10.3 (p=0.003)
Lactate during 8 min exercise	6.0 (p=0.02)	4.2 (p=0.04)
HOMA-IR	2.3 (p=0.14)	0.8 (p=0.38)
Behavioral RPE predictor model		
CES-D	4.5 (p=0.04)	3.2 (p=0.08)
HOMA-IR	2.5 (p=0.11)	1.5 (p=0.23)
Physiological lactate predictor model		
Heart rate during 8 min exercise	5.6 (p=0.02)	8.9 (p=0.0002)
T2DM diagnosis (yes/no)	6.2 (p=0.02)	1.6 (p=0.26)

Separate linear regression models were conducted for physiological predictors of RPE, behavioral predictors of RPE and physiological predictors of lactate during exercise at 30 W and at 35% VO_2_peak.

CES-D, Center for Epidemiological Studies Depression; HOMA-IR, Homeostasis Model Assessment of Insulin Resistance; RPE, Rating of Perceived Exertion; T2DM, type 2 diabetes mellitus.

### Behavioral effort-related variables

We found no significant difference in depressive symptoms or self-efficacy to perform physical activity by study group (table 1). In a multivariate regression model assessing predictors of RPE with adjustment for HOMA-IR, we found that depressive symptoms as measured by the Center for Epidemiological Studies—Depression (CESD)^39^ scale were associated with RPE at 30 W but not at 35% VO_2_peak. Depressive symptoms as measured by the PHQ-9^40^ scale were not predictive of RPE at either work rate.

## Discussion

We found that objective measures of exercise effort during submaximal exercise of lactate and heart rate during exercise were significantly greater in older women with T2DM compared to controls without diabetes. In addition, we identified higher levels of perceived effort in women with T2DM than in controls without diabetes, but these group differences in RPE were not statistically significant. Although RPE did not differ significantly between study groups, the RPE group differences observed at both 30 W and 35% VO_2_peak were ≥1 point—others have reported a 1-point difference to be clinically meaningful on the RPE scale.^44^ In addition, we found both heart rate and lactate during exercise to be significant predictors of RPE in the multivariate analysis, supporting the existing research that these three measures are all related markers of effort.^30^ ^45^ Taken together, these measures suggest that women with T2DM experience exercise, even at relatively low levels, to be more strenuous than their counterparts without diabetes. Exercise effort is an important barrier to physical activity because it is modifiable^11–13^ and the perception of more intense effort during exercise has been associated with lower levels of usual physical activity.^14–16^

As has been previously shown in adolescents and adults with T2DM, we found that mean levels of peak cardiorespiratory fitness were significantly lower in women with T2DM than in controls.^10^ ^20^ ^22^ ^23^ Similar to the finding of Wilkerson *et al*^23^ in older men, but different from our findings in younger adults,^10^ ^22^ the present study found no differences in mean τ_2_ levels in the T2DM group as compared to controls (table 1). Of note, the values of τ_2_ in our control participants were longer than those observed in prior studies of adolescent and premenopausal control participants,^22^ ^24^ ^32^ suggesting that the kinetic differences observed in younger T2DM may make them physiologically ‘older’ compared to their similarly-aged peers. Conversely, the lack of difference in subjects with T2DM versus controls in older populations (eg, Wilkerson *et al*^23^ and the present study) suggests that age-related changes in this response parameter may mask the independent effects of T2DM.

Few studies have compared RPE in people with T2DM to RPE in people without diabetes. The existing literature suggests that RPE differences by T2DM status are more likely to be apparent at absolute work rates than at relative work rates that account for the lower fitness levels exhibited in people with T2DM than controls.^9^ ^46^ In our prior study of premenopausal women, we assessed RPE at two absolute work rates of 20 W and 30 W during cycle ergometer exercise and found significantly greater RPE in women with T2DM when compared to both overweight controls and normal weight controls with no T2DM.^9^ Coquart *et al*^46^ studied overweight women with T2DM and overweight controls at a relative work rate of 100% ventilatory threshold (mean work rate in watts: T2DM: 41±14; overweight control: 43±11) and found no significant group differences in RPE (T2DM=13.7±2.3, overweight control=13.2±1.6). Consistent with the concept that RPE differences are more attenuated at relative work rates than absolute work rates, in the present study the RPE differences between T2DM women and controls were smaller at the relative work rate of 35% VO_2_peak than at 30 W, although the group differences were not statistically significant at either 30 W (p=0.08) or at 35% VO_2_peak (p=0.20).

After adjustment for insulin resistance in our population of overweight women with and without T2DM, we found that the objective markers of effort of plasma lactate and heart rate during exercise were very strong predictors of RPE, as has been observed in other healthy populations.^47^ A somewhat surprising finding was that RPE was more strongly associated with a diagnosis of hypertension than with heart rate and plasma lactate during exercise. This finding warrants confirmation in future studies to ensure that it is not spurious. Others have reported an association between glucose levels and RPE during high-intensity exercise of prolonged duration because the ‘depletion of carbohydrate fuel sources triggers muscular fatigue’.^46^ ^47^ We did not find an association between glucose levels during exercise and RPE, thus supporting the existing literature that energy substrates such as glucose do not appear to influence RPE at lower exercise intensities.^47^

Our findings add to the literature suggesting that lactate levels during rest and exercise are greater in people with T2DM than in their counterparts without diabetes. Previous studies of differences in lactate levels during exercise in people with T2DM versus controls have been conducted in younger women^10^ and middle-aged men,^48^ but we are not aware of other studies in older women with T2DM versus no diabetes. Resting lactate concentrations were greater in people with T2DM as compared to controls without diabetes in prior studies as well as the present study—elevated lactate at rest has even been recognized as a predictor of incident T2DM.^49^ ^50^ In prior studies by Regensteiner and Mogensen *et al*, the lactate concentrations during exercise were significantly greater in the T2DM group versus overweight control group, and the differences became larger at higher work rates. With moderate intensity exercise, lactate levels may modestly increase as we observed, and lactate dehydrogenase (LDH) activity increases and can offset/counter-balance hydrogen ion production in order to delay muscle fatigue.^51^ ^52^ Our observations of higher absolute lactate levels at rest and during exercise may indicate an alteration of the LDH complex and function in T2DM—or perhaps a different position on the LDH complex operating curve. We observed wide interindividual variance in Δlactate concentrations and were not powered to detect significance in these data. The wide interindividual variance in our observed Δlactate concentrations may relate partly to the effect of different LDH isoforms and partly to variance in exercise intensity perturbation. In other studies assessing metabolic predictors of exercise effort, absolute lactate concentrations during exercise have been used as RPE predictors rather than Δlactate concentrations.^30^ ^45^ A scientific rationale for using the absolute concentrations of lactate rather than Δlactate is that lactate is dynamically produced and metabolized at rest and during exercise and levels of metabolism are proportionally higher at greater lactate concentrations.^52–54^ Thus, the absolute lactate concentration provides a meaningful comparison of the absolute difference between lactate production and metabolism at any given time. The 2010 American Diabetes Association physical activity guidelines suggest that “affective responses to exercise may be important predictors of physical activity adoption and maintenance”.^3^ Perceptions of disproportionately greater effort during exercise may be one reason that people with T2DM are less active than their counterparts without T2DM, as perceptions of exercise effort over a certain threshold worsen the affective response to exercise and are linked to lower levels of leisure physical activity.^14–16^ Our effort measures were obtained at work rates that are less intense than many activities of daily living. Since people tend to prefer physical activities with an intensity in the 11–14 range,^55^ ^56^ our findings provide some additional support to existing concerns that overweight, sedentary individuals with and without T2DM may avoid activities of daily living because they are perceptually too difficult and hence unpleasant. For example, our study population of overweight, sedentary women that reported mean RPE levels in the 10–12 range during cycle ergometry at 30 W (approximately 3 METs of intensity) would be expected to experience a much higher RPE while walking up a flight of stairs that represents approximately 10 METs of intensity.^57^ Thus, activities of daily living that require greater intensity activity, such as walking up stairs or walking at a faster pace to keep up with others, may be avoided for individuals such as those we studied. In keeping with this theory, a prior barrier reported by people with T2DM is the inability to keep pace with others who do not have diabetes.^17^ A recent review article suggested that practitioners balance goals for their patients to conduct regular exercise in a range of intensity that will allow for physiological improvement and the need to make physical activity ‘palatable’ in an intensity range that is acceptable to the individual.^58^ An important take-home point for clinicians is to encourage patients to be physically active at a pace that is personally comfortable as this tends to be associated with both good adherence and a good physiological fitness response.^58^ ^59^

The strengths of this study include the assessment of effort during exercise by both subjective (RPE) and objective methods (plasma lactate and heart rate), as well as assessments of both physiological and behavioral predictors of exercise effort. The cross-sectional nature of the study prevented us from drawing any causal inferences for the statistical associations that we observed between exercise effort and the predictors of effort. Although the control subjects did not meet a diagnosis of prediabetes by the prevailing criteria of the time, a subset would have met the current criteria for prediabetes by a HbA1c of 5.7–6.4%. The inclusion of control subjects with prediabetes may have diminished the differences in exercise effort observed between the study groups. Another limitation of these findings is that the duration of exercise bouts assessed was only 8 min; evaluating the effort response to longer bouts of duration would also be important to examine in future studies.

In summary, we found what others have reported to be clinically meaningful differences in RPE^44^ and statistically significant differences in lactate and heart rate during low-to moderate-intensity exercise in postmenopausal women with T2DM, as compared to their counterparts without diabetes. The group differences in RPE were not as large as we observed in our prior study of younger women,^9^ suggesting that future studies in older adults should use a more conservative estimate of effect size than is appropriate in younger women. It is possible that the effects of aging may influence T2DM-related exercise impairments. Greater perceived exertion is a modifiable and thereby targetable end point. Therefore, methods to reduce perception of work effort in T2DM should be sought in order to facilitate regular physical activity in people with T2DM. Improving physical activity would also improve the premature disability and mortality experienced by people with T2DM.
